# Feasibility of a Yoga Intervention in an Inpatient Limb Loss Rehabilitation Program

**DOI:** 10.33137/cpoj.v7i1.43896

**Published:** 2024-10-24

**Authors:** A.L Mayo, B Cheung, J Li, S Jean, A Vijayakumar, S.L Hitzig, R Simpson

**Affiliations:** 1 St. John’s Rehab Research Program, Sunnybrook Research Institute, Sunnybrook Health Sciences Centre, Toronto, Canada.; 2 Temerty Faculty of Medicine, University of Toronto, Toronto, Canada.; 3 Department of Medicine, University of Montreal, Montreal, Canada.; 4 Department of Occupational Science and Occupational Therapy, Temerty Faculty of Medicine, University of Toronto, Toronto, Canada.; 5 Toronto Rehabilitation Institute, University Health Network, Toronto, Canada.

**Keywords:** Yoga, Qualitative, Survey, Pain, Amputation, Rehabilitation, Lower Extremity Amputation

## Abstract

**BACKGROUND:**

Limb loss is a life-changing event, which may be associated with limited mobility, pain, and low mood. Yoga interventions have been found to be beneficial for improving emotional wellness and pain in other patient populations. The benefits of including yoga in limb loss rehabilitation have not been well studied.

**OBJECTIVE:**

The purpose of this study was to determine if an adaptive yoga program would be suitable for individuals with newly acquired limb loss in a rehabilitation program.

**METHODOLOGY:**

A yoga video was co-designed by rehabilitation clinicians and a limb loss patient partner certified in yoga instruction. Surveys were used to collect patients’ socio-demographics and previous yoga experience. Participants completed a therapist guided group yoga video session, and then given online access to practice independently. Post-yoga participation surveys and qualitative interviews were conducted with patients to determine acceptance and feasibility of the yoga intervention.

**FINDINGS:**

Twenty-four participants with lower limb amputation(s) were approached to participate. The majority of participants (63%) had dysvascular-related amputations. Nineteen out of 24 recruited patients (79%) completed the yoga video session and the pre-yoga survey. Sixteen out of 19 participants completed the post-yoga survey, and eight also completed a qualitative interview. Five had previously undertaken yoga but rated themselves as novices. All participants felt that yoga was beneficial, easy to complete, and should be included in rehabilitation. Participants found yoga to be relaxing and some noted reduction in pain. Most preferred to do yoga in a group. Five out of eight patients (63%) interviewed continued to do the yoga video independently in hospital and post-discharge. Challenges with the yoga intervention included lack of a quiet yoga space, and dedicated time given other appointments/priorities.

**CONCLUSION:**

Yoga was widely accepted by the inpatient limb loss population. Yoga may complement traditional limb loss rehabilitation by providing patients a relaxing experience; however, further research is needed.

## INTRODUCTION

Lower extremity amputation (LEA) is associated with poor balance, decreased physical strength, and decreased mobility, as well as high rates of depression, and social isolation.^1–6^ Similar to global trends,^7,8^ the leading cause of LEA in Canada are dysvascular in nature due to complications of peripheral arterial disease (PAD) and/or diabetes mellitus (DM).^9–11^

Other causes of LEA (non-dysvascular) include trauma, cancer or non-diabetes/PAD related infection and/or ischemia.^12–15^ Patients with dysvascular LEA often have a high number of co-morbidities,^16^ which requires them to adopt a chronic condition management approach while adapting to life with a new physical disability.^17^ In particular, residual limb pain and phantom limb pain (PLP) are commonly experienced post-amputation,^18–20^ for both dysvascular and non-dysvascular LEA (e.g., trauma, cancer, etc.), and can further reduce physical function and wellbeing.^21–23^ As a result of reduced mobility and health challenges, individuals with LEA have high rates of sedentary lifestyles,^24^ which can exacerbate their chronic health conditions, such as heart disease, PAD and DM; thereby elevating their risk for mortality.^25^

In Canada, limb loss rehabilitation programs traditionally include physiotherapy, occupational therapy, and/or prosthetic training.^9^ Inpatient rehabilitation programs are typically accessed by patients with newly acquired limb loss and/or for first time training with a prosthesis.^9,17^ It is known that patients with limb loss have high rates of adjustment disorder, depression and/or anxiety in the first months following amputation when they are accessing rehabilitation services,^4^ and that patients often feel that their mental health needs are not prioritized.^26^ Consequently, there is a need for wellness programs in Canadian limb loss rehabilitation programs, which are currently lacking.^4,26^

Yoga has been found to be a promising intervention to improve health and wellness that can be adapted for people with physical disabilities. It has been found to improve mood and pain in a variety of patient populations, such as those with fibromyalgia, multiple sclerosis, and spinal cord injury.^27–30^ Yoga is an ancient practice with a focus on physical, mental, emotional, and spiritual health.^31^

Developed in India, yoga has eight domains or “limbs,” which consist of yama (universal ethics), niyama (individual ethics), asana (physical postures), pranayama (breath control), pratyahara (control of the senses), dharana (concentration), dhyana (meditation), and samadhi (bliss).^31^ Most healthcare yoga interventions have focussed on use of physical postures,^31^ breath control,^32^ and/or meditation.^33^ Qualitative reports of different patient groups who have participated in yoga often share positive reactions to the intervention,^34–36^ and that it had some advantages to other forms of exercise (e.g., less fatiguing, less discomfort).^33^

Adaptive yoga programs for individuals with limb loss exist^37^ but its benefits have not been well studied, nor has yoga been incorporated regularly into limb loss rehabilitation programs. Given the high rates of sedentary lifestyles, pain, and psychological distress post-limb loss, yoga may be a promising intervention that can be introduced early in the rehabilitative process. As well, people with limb loss are known to have difficulty accessing community services, such as exercise programs due to lack of transportation, financial costs, and/or lack of suitable facilities.^38,39^ Hence, Adaptive yoga may offer patients with an exercise option they can pursue if other factors (i.e., income, transportation) create barriers to leisure-time physical activity.

The original purpose of this study was to determine if a yoga for limb loss in-person program could be incorporated into a LEA inpatient rehabilitation program. However, due to the COVID-19 pandemic and episodes of restricted group exercise, the focus of the study was shifted to determine the feasibility and acceptability of using an online adaptive yoga video intervention among patients with LEA. We hypothesised that individuals with LEA would be interested in participating in an online yoga video program, and that it would be an acceptable addition to traditional limb loss rehabilitation programs.

## METHODOLOGY

A mixed methods study design was used to test the feasibility and acceptability of an adaptive online yoga intervention for inpatient rehabilitation patients with newly acquired limb loss. The main research question was to determine if an adaptive virtually-delivered yoga program for limb loss is feasible to implement in a rehabilitation inpatient setting, and to determine if the program would be well-received by participants. Post-yoga Likert scale intervention surveys were used to determine participant acceptance of, and experiences with the adapted yoga program. Semi-structured interviews were conducted to explore participant thoughts on perceived benefits, what is needed to improve the program, and to gather other suggestions for making the yoga intervention more accessible to the wider limb loss community.

### Participants

The inclusion criteria for the study were English-speaking adult patients with LEA of any etiology (dysvascular, trauma, cancer, etc.) admitted to the Inpatient Amputee Rehabilitation Unit at Sunnybrook St. John’s Rehab Hospital in Toronto, ON, Canada. To take part, participants were required to be medically cleared physically and cognitively to be able to participate in the adaptive online yoga intervention by the unit’s physical medicine and rehabilitation physician (Physiatrist). In addition, participants needed to have internet access in their homes, a device that would enable them to access the adapted online yoga intervention, and were able to undergo the study assessments. This study received ethics approval by the Research Ethics Board of the Sunnybrook Health Sciences Centre (REB #5227).

### Adaptive Yoga Intervention

The yoga intervention was designed by our team of physiatrists with expertise and training in yoga and LEA (Mayo A.L, Simpson R), as well as by a physiotherapist (Cheung B) and an occupational therapist (Li J) with LEA expertise. The basis for the program was established by reviewing Marsha Danzig’s Yoga4Amputee (https://www.yogaforamputees.com) instructor training materials,^37^ and by consulting with a study partner, who is a person with LEA (Hauer, L) and a certified yoga instructor. To accommodate individuals with new LEA who may not yet have a prosthesis, a chair-based yoga program was created. The created video was 45 minutes, and the instructor for the video yoga session was our patient partner with LEA. The yoga video was made available online on a YouTube channel, which was only accessible to those who were provided a link for viewing. The physiotherapist (Cheung B) and occupational therapist (Li J) on our study team completed online training provided by Yoga for Amputees (https://www.yogaforamputees.com/classestraining) to learn the yoga exercises. These therapists provided orientation to study participants, and supervised participants who took part in a group video session. Participants completed the yoga video session in groups of 2-4 during their inpatient amputee rehabilitation admission. Once the group video session was completed participants were given the YouTube link so they could continue to participate in yoga independently.

### Procedure

**Figure1:**
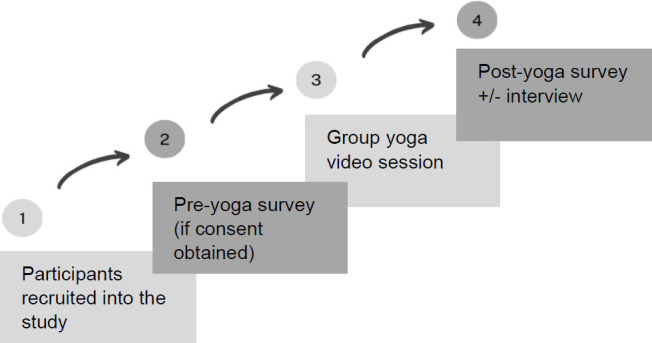
Study protocol flow chart.

Admitted patients with LEA to St. John’s Rehab Hospital who met the inclusion criteria were informed about the study by a member of their rehab care team (therapist or physician). If the participants wanted to learn more, the study coordinator followed-up and obtained informed consent for those participants who agreed to participate. Twenty-four participants were recruited into this study between August 2022 and June 2023. A study flow-chart is illustrated in **Figure1**.

**Figure 2:**
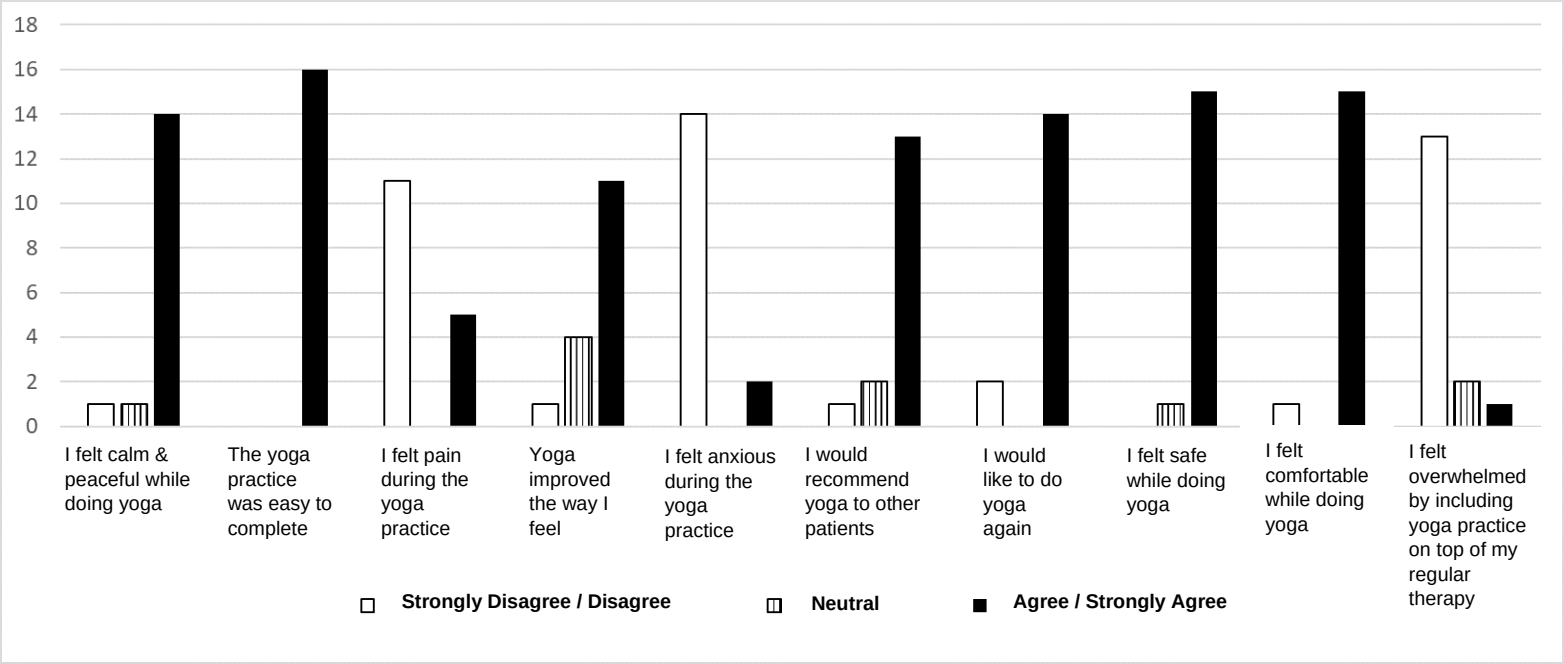
Post-yoga survey results (n=16 participants).

Prior to completing the yoga session, participants completed a Likert-scale survey comprised of 10 questions that collected data on socio-demographics, previous yoga experiences, and digital literacy (**Figure 2**). To understand more in-depth the individual experiences of participants with the yoga intervention, participants were requested to take part in a post-yoga survey, and in a semi-structured interview. The interview incorporated the following five questions:

1. Why were they interested in participating in the program?
2. What concerns, if any, did they have about participating in an online yoga intervention?
3. What aspects they enjoyed or did not like?
4. Suggestions for improvement?
5. Any other insights about promoting yoga to the limb loss community?

These interviews were conducted over phone or by Zoom and ranged from 10-30 minutes. Interviews were then transcribed verbatim for narrative analysis.

### Analysis

Descriptive statistics and frequencies were used to analyze the data. To understand acceptability and feasibility of the adaptive online yoga intervention, the rate of yoga participation was calculated, which included the ability of participants to complete the video in the group session at the inpatient rehabilitation setting, and independent use of the yoga video post group session was calculated. For the interview data, a narrative research approach^40–42^ was taken to highlight participant experience and feedback regarding their attitudes about yoga, its’ perceived benefits, and recommendations for improvement. Study team members (Vijayakumar A, Mayo A.L) coded responses and identified themes from the interviews. Given that the interviews were relatively short (10 – 30 minutes), a descriptive narrative summative approach was determined to be sufficient to obtain critical insights about the yoga intervention. To help illustrate the main findings of the interview data, illustrative quotes with key participant descriptors are provided.

## RESULTS

Of the twenty-four participants who were deemed eligible and approached to participate in the study, 19 agreed to participate in the yoga intervention (79% enrollment rate). All of the participants were those with a newly acquired LEA within the past three months, with one patient who was admitted for a second amputation with history of a contralateral amputation the year prior. Seven (37%) had a traumatic etiology, and the rest (63%) had a dysvascular etiology. Four participants were female (21%) and 15 (79%) were male. Participants’ ages ranged from 25 to 85 years old, with an average age was 58.9 years (SD=18.9). Only four of the participants (21%) had participated in yoga before the study, and all these participants rated themselves as novice level yoga participants. In terms of digital literacy, the majority self-rated themselves as having good levels of literacy (68%), with only two rating themselves as being very good or excellent (11%), and the rest stating it was fair or poor (21%). The complete demographics of the participants, self-rated technology skills and yoga experience are outlined in **Table 1**.

**Table 1:** Demographics of the study population.

Age	Sex	Level of education	Etiology	Level of amputation	Technology Skills	Yoga experience	Yoga level
59	Male	College	Trauma	BKA^*^	Good	No	N/A
25	Male	Somecollege	Trauma	BKA	Good	No	N/A
46	Female	High school	Trauma	AKA^**^	Good	No	N/A
68	Male	College	Dysvascula	BKA	Very Good	No	N/A
39	Male	Some college	Trauma	BKA	Good	Yes	Novice
57	Female	High school	Trauma	AKA	Fair	Yes	Novice
68	Female	Some high school	Dysvascula	AKA	Poor	No	N/A
31	Male	High school	Dysvascula	BKA	Good	No	N/A
81	Male	Some high school	Dysvascula	AKA	Fair	No	N/A
85	Male	High school	Dysvascula	AKA	Good	No	N/A
69	Male	College	Dysvascula	BKA	Fair	No	N/A
28	Male	High school	Trauma	AKA	Good	Yes	Novice
58	Male	Some high school	Dysvascula	BKA	Good	No	N/A
74	Male	University	Trauma	AKA	Good	Yes	Novice
50	Male	High school	Dysvascula	AKA	Good	No	N/A
74	Male	High school	Dysvascula	BKA	Good	No	N/A
83	Male	High school	Dysvascula	BKA	Good	No	N/A
48	Male	College	Dysvascula	BKA	Excellent	Yes	Novice
76	Female	University	Dysvascula	AKA/BKA	Good	No	N/A

* Below-the-knee amputation (BKA)

** Above-the-knee amputation (AKA)

Of the 19 participants that participated in the adaptive yoga intervention, 16 (84%) completed the post-yoga survey (**Appendix A**), while 3 were unable to complete it due to being discharged from inpatient rehabilitation or declined to complete. Post-yoga survey results are illustrated in **Figure 2**. The results of the post-yoga survey report that the majority participants found yoga to be a positive calming experience. All but one yoga participant (95%) were comfortable doing yoga and only 2 of the 16 (13%) respondents stated they did not wish to do yoga again. All participants found the video easy to complete.

Eight (42%) yoga participants completed post-yoga semi-structured interviews in addition to the post-yoga survey via phone or Zoom. Key themes that were identified from the transcripts were openness to yoga, calming/relaxing benefits, peer support, and need for a dedicated yoga space and time in rehabilitation.

### Expectations and Openness to Yoga

Most of the participants did not have any pre-set expectations of taking part in yoga, and indicated they joined to just learn more about it and how it may help them to stay more active. For instance, one person stated: “*I didn’t have any expectations or hopes, to be honest, I was just trying it out.*” (Male, 25 years, Left BKA), with others commenting:

“*I just want to experience what yoga is*.” (Male, 57 years, Right AKA)

“*So, I was actually very open to it. I feel it helped that I had a good relationship with the roommate at the time I had, so it was easier to get involved, so it made it even more enjoyable and comfortable to go to, because you had a familiar companion and a friend.*” (Male, 39 years, Left BKA).

Once tried, however, participants stated they would like more yoga sessions to participate in during rehabilitation.

### Therapeutic Benefits

In terms of the perceived therapeutic benefits, all eight participants found the yoga session calming. One patient who had experienced recent trauma found the session to be relaxing where he stated: “*Honestly, I liked the way it went. Just in a room, quiet, I was at peace*.” (Male, 25 years, Left BKA). As well, participants felt it complimented their formal rehabilitation by offering gentler exercises, stretching, and teaching breathing techniques. One person commented that compared to their physiotherapy sessions, yoga was not painful at all. Other specific aspects that participant enjoyed were the stretching:

“*Physically, I love the stretches. I love the way I feel limber, but comfortable. Because when you’re going through this process, you’re using other muscles to compensate for your balance, just your motion, how you do day-to-day stuff. You’re using different parts of your back muscles that you don’t use daily, so you get tight and crampy. So, the stretching from the yoga was great for that*.” (Male, 74 years, AKA and BKA)

Overall, all the participants enjoyed the yoga video, felt safe completing the video, and were happy they participated in the program.

### Group in Setting with Peers

Participants appreciated having a peer limb loss instructor for the video since they felt it modeled ways on how they could move with their amputation. In general, the ability to connect with peers was highly valued, with six of the eight (75%) participants rating they would benefit the most from yoga doing it in a group setting. One person shared: “*Socially. Yes, I knew the guys in there. Maybe I met a couple guys in there that weren't there before. So, yes, there are benefits of that*” (Female, 74 years, Left BKA). Overall, it provided a number of social opportunities to patients, and highly beneficial for receiving peer support. Importantly, one participant (Male, 25 years, BKA) commented that his post-traumatic stress disorder (PTSD) made him unable to focus on yoga if he was alone.

### Yoga Post-Discharge

Five out of the eight (63%) interviewed participants continued to do the yoga video after the group session while still in inpatient rehabilitation and at home. Participants completing the video independently were able to access the video without issues. Two of the home participants felt yoga was better while admitted to rehabilitation because there were more distractions at home, and no one to supervise them or do yoga with at home.

“*That's the difficult part because now you've changed environments. Unless people really find it beneficial to them and necessary, they're not going to follow. Perfect example, right here. And that's the way it will go unless they have had enough sessions that they see how the real long-term benefit of this. One, two or three sessions*.” (Female, 74 years, Right AKA).

Hence, there were mixed findings regarding participants’ willingness to continue with yoga once discharged home, and that some participants reported they may be more inclined to participate in yoga more independently if they were reminded or coached to do so.

### Feedback and Recommendations

Many participants stated the chosen yoga room (unit patient education room) was not ideal for the intervention. One person commented: “*Like I said, probably a proper room, to put the people on a mindset, like rest. Like for the people feel more rest and focus on the class*.” (Male, 28 years, Right AKA). As well, all eight interviewed participants’ desired yoga to be a scheduled session in regular rehabilitation programming. Many participants wanted to do scheduled yoga at least once a week.

“*Two times a week was pretty cool. It wasn’t a big group, so I think it worked for that. I think as a group gets bigger, you can have an A group or B group, like a morning group, afternoon group. I don’t know, something like that*.” (Male, 39 years, Left BKA).

Other suggestions by participants were to have pamphlets or information packages to give to patients before they enroll, and that a mix of in-person and online sessions would be preferred, with one person commenting: “*Well, I'm still old school, but I still like the in person and it's more personal. Even if you got six or eight people that are with you, it's more personal if you got an instructor there*.” (Female, 74 years, Right AKA). As well, issues of digital literacy and access were raised as potential barriers.

## DISCUSSION

The purpose of the present study was to explore the feasibility and acceptability of an adapted online yoga video program. Based on our findings, it appears there was a high degree of interest in exploring yoga given that 79% of approached participants wanted to learn more. As well, despite our participants having little or no experience with yoga, they were enthusiastic about participating in the intervention based on our survey and qualitative data; with many of them reporting to find it beneficial for their wellbeing. This finding is similar to other studies exploring yoga in other patient populations. For instance, one study that piloted an adapted yoga program for spinal cord injury patients had qualitative reports by patients stating they had high satisfaction with the program, that it helped them with experiences of the self and relaxation, and helped with pain relief.^28^ Similar to other studies on other populations where patients noting yoga had advantages over other forms of exercise,^33^ our participants’ commented that participating in yoga was less strenuous than other activities typical of inpatient rehabilitation (e.g., physiotherapy) and it may be that relaxation is a modality underutilized in current rehab programming for LEAs.

Of the patients interviewed, 5/8 (63%) continued the yoga intervention practice beyond the study intervention session. Frequent practice of yoga has been associated with physical, emotional, and mental health benefits.^43^ Studies exploring the impact of yoga often do so over a series of sessions. For instance, a recent randomized controlled trial that tested the benefits of yoga in a traumatic LEA population found that those (n=24) who took part in the 18 week yoga intervention (daily 30 minute yoga session; including unsupervised sessions) yielded significant improvements in overall quality of life, as well as in physical, psychological and environmental domains of wellbeing compared to a group receiving usual prosthetic and rehabilitation treatments without yoga (n=23). These effects were detected as early as six weeks.^44^ Our yoga intervention showed promise in being a suitable adjunct activity to inpatient rehabilitation, but our participants indicated they would like more frequent yoga sessions. Similar findings for more frequent sessions have been noted in other yoga studies.^36,43^ Integration of yoga practice into regular rehabilitation programming would appear to benefit participants but a suitable quiet space, yoga content, and scheduling of yoga around other rehab activities would be needed.

Data on the benefits of yoga for dysvascular LEA patients, however, is needed, and the nuances and appropriateness of yoga for trauma populations requires further attention. For instance, one of our participants with a traumatic etiology noted that doing yoga on his own triggered his PTSD. PTSD is a common issue following major trauma, and is associated with a variety of symptoms, including intrusions (e.g., unwanted flashbacks), engaging in avoidant behaviors to avoid trauma reminders, negative alterations in mood and cognition, and hypervigilance.^45^ There is evidence that mindfulness-based approaches (encouraging of fostering nonjudgment and acceptance of one’s thoughts and emotions as they occur in the present moment),^46^ which yoga interventions sometimes integrate through the meditation component, are not well-suited for trauma populations if not properly introduced or monitored. For some persons with PTSD, mindfulness-based approaches may lead to increased distress, especially individuals who are prone to flashbacks, rumination, or are easily triggered by their trauma memories.^47^ Thus, although adapted yoga holds promise for people with LEA, like Mindfulness-based approaches, it too may need further adaptation to ensure it is ‘trauma sensitive’.

Not surprisingly, participants were interested in undertaking yoga in a group and appreciated having the online video being led by someone with limb loss. In one qualitative study exploring facilitators and barriers to physical activity, participants (N=33) commented about the importance of social support and being surrounded by peers with a shared mutual experience of the impact of limb loss.^38^ Hence, participating in a group of peers that was led, albeit asynchronously, by a peer was likely a motivating factor for wanting to take part in the intervention.

### Limitations

There are several limitations to the present study. First, the original purpose was to do an in-person yoga program at our inpatient setting. Unfortunately, our study was set to commence just as the COVID-19 pandemic occurred, which required us to delay the start of the program, and then to modify it to minimize the spread of potential infection. This re-adjustment led to the creation of an online video whereby our team was able to introduce the yoga intervention by a yoga trained physiotherapist and occupational therapist, which was then pursued by participants on their own accord. Unfortunately, we were only able to create a 45-minute session video, and did not collect data on efficacy of the program. This was not pursued due to our initial desire to create a suitable adapted yoga program that had basis for the limb loss population.^37^ As well, our emphasis was on feasibility and acceptability, and therefore we focused on those metrics as the starting point for the development of the intervention. Our data indicates that an adapted online yoga video was feasible to implement in our inpatient setting, but its’ broader feasibility cannot be determined based on our available data. Despite the limitations of the study, it does appear that yoga was deemed acceptable by our participants through the ratings on the survey and by their qualitative reports.

### Future work

Future work should explore clinical efficacy via a more robust controlled trial for LEA populations as well as hybrid or tele-rehabilitation models of delivery. As noted, people with LEA fail to participate in sufficient levels of physical activity, which increases their risk for the development of cardiovascular disease, and mortality.^24,48,49^ There is evidence that tele-rehabilitation approaches can serve to improve yoga adherence and is clinically effective in a variety of rehabilitation patients, which may help to maintain long-term benefits and engagement in physical activity.^50–52^ Studies evaluating Yoga-based interventions should consider how to facilitate their uptake while in clinic to ensure safe practices are taught to patients while a tele-rehabilitation or app-based component may enable patients to continue with yoga once home, which may provide a source of activity to those who face numerous barriers to accessing exercise in their communities. As well, a more robust study should be undertaken to further explore the nuances of the benefits of yoga post-limb loss.

## CONCLUSION

An adaptive yoga video for people with LEA was widely accepted by the inpatient population in this study. Yoga may complement traditional limb loss rehabilitation, which can enable patients to better adjust early post-amputation while providing a physical activity that is relaxing, and that holds a number of benefits to their wellbeing. The introduction of an adapted yoga intervention while in-hospital that is paired with an online video they can use once discharged from hospital may contribute to patients’ wanting to continue in the engagement in this beneficial mind-body intervention. However, more research is needed to explore implementation considerations and suitability of this intervention for different sub-types of patients, as well as to determine clinical efficacy.

## DECLARATION OF CONFLICTING INTERESTS

The authors have no declared conflicts of interest.

## AUTHORS CONTRIBUTION

- **Amanda Mayo**: conceived the study design, obtained the funding for the project, worked to design the yoga intervention with a patient partner with expertise in yoga, oversaw the intervention, analyzed the data, wrote and revised the manuscript.
- **Betty Cheung**: obtained the funding for the project, worked to design the yoga intervention with a patient partner with expertise in yoga, oversaw the intervention, and analyzed the data.
- **June Li**: obtained the funding for the project, worked to design the yoga intervention with a patient partner with expertise in yoga, oversaw the intervention, and analyzed the data.
- **Stephanie Jean**: worked to design the yoga intervention with a patient partner with expertise in yoga and edited the manuscript.
- **Abirami Vijayakumar**: coordinated the study, recruited participants, collected data, and analyzed the data.
- **Sander Hitzig**: conceived the study design, obtained the funding for the project, wrote and revised the manuscript.
- **Robert Simpson**: conceived the study design, obtained the funding for the project, and worked to design the yoga intervention with a patient partner with expertise in yoga.

All authors reviewed the manuscript and approved the final version.

## SOURCES OF SUPPORT

This research study was funded by the Sunnybrook Health Sciences Centre Practice Based Research and Innovation Seed Grant.

## Appendix A: Likert Survey Post-Yoga Session

1) I felt calm and peaceful while doing yoga
  **1-**Strongly disagree; **2-**Disagree; **3-**Neither agree nor disagree; **4-**Agree**; 5-**Strongly Agree
2) The yoga practice was easy to complete
  **1-**Strongly disagree; **2-**Disagree; **3-**Neither agree nor disagree; **4-**Agree**; 5-**Strongly Agree
3) I felt pain during the yoga practice
  **1-**Strongly disagree; **2-**Disagree; **3-**Neither agree nor disagree; **4-**Agree**; 5-**Strongly Agree
4) Yoga improved the way I feel
  **1-**Strongly disagree; **2-**Disagree; **3-**Neither agree nor disagree; **4-**Agree**; 5-**Strongly Agree
5) I felt anxious during the yoga practice
  **1-**Strongly disagree; **2-**Disagree; **3-**Neither agree nor disagree; **4-**Agree**; 5-**Strongly Agree
6) I would recommend yoga to other patients
  **1-**Strongly disagree; **2-**Disagree; **3-**Neither agree nor disagree; **4-**Agree**; 5-**Strongly Agree
7) I would like to do yoga again
  **1-**Strongly disagree; **2-**Disagree; **3-**Neither agree nor disagree; **4-**Agree**; 5-**Strongly Agree
8) I felt safe while doing yoga
  **1-**Strongly disagree; **2-**Disagree; **3-**Neither agree nor disagree; **4-**Agree**; 5-**Strongly Agree
9) I felt comfortable while doing yoga
  **1-**Strongly disagree; **2-**Disagree; **3-**Neither agree nor disagree; **4-**Agree**; 5-**Strongly Agree
10) I feel overwhelmed by including yoga practice on top of my regular therapy
  **1-**Strongly disagree; **2-**Disagree; **3-**Neither agree nor disagree; **4-**Agree**; 5-**Strongly Agree
